# Pathogen-triggered changes in plant development: Virulence strategies or host defense mechanism?

**DOI:** 10.3389/fmicb.2023.1122947

**Published:** 2023-02-15

**Authors:** Feng Kong, Li Yang

**Affiliations:** Department of Plant Pathology, University of Georgia, Athens, GA, United States

**Keywords:** plant-pathogen interaction, plant development, virulence, fitness, disease symptom

## Abstract

Plants, as sessile organisms, are constantly exposed to pathogens in nature. Plants rely on physical barriers, constitutive chemical defenses, and sophisticated inducible immunity to fight against pathogens. The output of these defense strategies is highly associated with host development and morphology. Successful pathogens utilize various virulence strategies to colonize, retrieve nutrients, and cause disease. In addition to the overall defense-growth balance, the host-pathogen interactions often lead to changes in the development of specific tissues/organs. In this review, we focus on recent advances in understanding the molecular mechanisms of pathogen-induced changes in plants’ development. We discuss that changes in host development could be a target of pathogen virulence strategies or an active defense strategy of plants. Current and ongoing research about how pathogens shape plant development to increase their virulence and causes diseases could give us novel views on plant disease control.

## 1. Introduction

In nature, plants and their pathogens are in a continuous coevolutionary arms race (Burdon and Thrall, 2009). Plants, as sessile organisms, are attacked by various pathogens including bacteria, fungi, oomycetes, and nematodes. They evolved efficient constitutive and inducible innate immune responses to detect pathogens and defend themselves from disease (Bigeard et al., 2015). In general, plants are resistant to most pathogens because of preformed physical barriers (e.g., cuticle and cell wall; Hamann, 2012), and constitutive chemical defenses (e.g., antimicrobial compounds; Osbourn, 1996). These physical barriers are often dependent on the developmental stages of a plant host and/or present on specific organs (Smith, 2001; Ingram and Nawrath, 2017; Wolf, 2022). For example, secondary cell walls are strengthened in fully mature tissues (Zhong and Ye, 2015; Meents et al., 2018); cuticle layers are thicker in adult plants (Yeats and Rose, 2013; Budke and Goffinet, 2016; Ingram and Nawrath, 2017).

Two intertwined inducible immune responses protect plants from invading pathogens. On the cell surface, a group of membrane anchored or associated pattern recognition receptors (PRRs) recognize conserved microbial elicitors, including bacterial flagellin (Hayashi et al., 2001), elongation factor Tu (EF-Tu; Zipfel et al., 2006), and fungal cell wall components like chitin and polysaccharides (Kaku et al., 2006; Yin et al., 2016), which triggers a collection of immune responses called pathogen-associated molecular patterns (PAMP)-triggered immunity (PTI; Bigeard et al., 2015; Tang et al., 2017; Saijo et al., 2018). Successful pathogens can secrete effector proteins, hormone mimics, toxins, and other small molecules to compromise the host plants immune system, or alter other cellular processes (Ngou et al., 2022). Many effectors can increase pathogen virulence by countering PTI, which causes effector triggered susceptibility (ETS; Guttman et al., 2006; Jones and Dangl, 2006; Kwi-Mi et al., 2008). The functions of pathogen effectors have been widely discussed and reviewed (Deslandes and Rivas, 2012; Dou and Zhou, 2012; Howden and Huitema, 2012; Ngou et al., 2022). Within a cell, nucleotide binding-leucine-rich repeat (NLR) proteins act as intracellular receptors to detect effectors and activate effector-triggered immunity (ETI; Jones et al., 2016). PTI components are required for the full activation of ETI, and ETI also enhances PTI responses (Naveed et al., 2020; Tena, 2021; Yuan et al., 2021; Chang et al., 2022). Both PTI and ETI can induce a series of immune responses, such as stomata closure (Zhang et al., 2008; Sawinski et al., 2013; Yuan et al., 2021), reactive oxygen species burst (O’Brien et al., 2012), the hypersensitive response (Mur et al., 2008), the production of antimicrobial compounds and defense-related proteins (Bednarek, 2012), and mitogen-activated protein kinases (MAPKs) cascades activation with ETI often manifests in the faster and stronger forms (Meng and Zhang, 2013).

Given the essential function of PTI and ETI in defense, components of these immune signaling pathways are under tight spatial-temporal regulation. Plants balance development and defense to ensure resource allocation, quick adaptation to changing environment, and eventually successful reproduction (Huot et al., 2014). Mis-regulation of their expression level or function may lead to hyper-activation of immune response and eventually stunted growth or cell death (Li et al., 2020). Arabidopsis mutants with constitutively activated defense suffer from autoimmune symptoms including dwarfism and lesion formation (Rodriguez et al., 2016; van Wersch et al., 2016). High disease resistance in some crop varieties can also be associated with reduction in yield, a phenomenon known as yield penalty (Brown, 2002). Spatiotemporal regulation of key immune components is essential to prevent misfiring. For example, the response to bacterial derived elicitor flg22, a 22 amino acid peptide derived from flagellin, is confined to the root cap and transition/elongation zone in Arabidopsis (Emonet et al., 2021). Such immune response is further gated by a co-incident wound signaling to ensure the proper development of root in a microbial-rich environment (Zhou et al., 2020). Defense responses can also be differentially activated at distinct stages of host development. In Arabidopsis and tobacco, the old plants accumulate more Salicylic Acid (SA) and exhibit stronger SA responses than young plants (Carella et al., 2014; Wilson et al., 2017). Similar age-dependent resistance is associated with early seedling development, maturation of individual organs, or flowering (Kus et al., 2002; Rusterucci et al., 2005; Wilson et al., 2017). Taken together, plant development and immune response are highly coordinated by intrinsic molecular networks.

On the other side, pathogens can utilize multiple strategies to colonize plants, obtain nutrients, enhance susceptibility, and cause disease (Stavrinides et al., 2008). Pathogen infection often affects the growth and developmental processes of the host (Kwi-Mi et al., 2008). An interesting example is the formation of various gall structures when a plant is colonized by insects or fungi (Harris and Pitzschke, 2020). Most plant-formed galls are pathogens’ feeding sites to accommodate infection including some of the most studied pathogens *Rhodococcus fascians, Pseudomonas savastanoi, rust fungi, Agrobacterium tumefaciens, root cyst nematodes,* and *gall midges* (Harris and Pitzschke, 2020). Pathogen-induced changes in plant development can be an outcome of the pathogen’s virulence strategies or the active plant defense strategy upon pathogens infection. Here, we review recent advances in understanding the molecular signaling of pathogen-triggered changes in the development of specific tissues/organs and discuss the potential impact of these developmental changes in host-pathogen interactions. Excellent reviews on the overall balance between defense and growth/biomass can be found here (Huot et al., 2014; Figueroa-Macías et al., 2021; He et al., 2022). Here, we listed various aspects of pathogen effects on plant growth and development as shown in Table 1.

**Table 1:** Examples of pathogen-induced changes in plant growth and development with known molecular mechanisms.

Pathogens	Molecular mechanisms	References
*Pathogen modulates host hormone signaling*		
*Meloidogyne incognita and Heterodera schachtii*	Synthesis hormone auxin and induce plant cell reprogramming; induce a feeding cell complex or form a giant cell	Niebel et al. (1994), Siddique et al. (2015), and Oosterbeek et al. (2021)
*Heterodera schachtii*	Secret effector protein 19C07; target the Arabidopsis LAX3 auxin import protein; trigger the cell wall hydrolysis to stimulate syncytium development and lateral root emerge	Lee et al. (2011)
*Heterodera schachtii*	Secret effector 10A07 and interact to plant kinase (IPK) and the auxin regulator protein IAA16 preventing it from repressing auxin response genes, stunted shoots, and roots	Hewezi et al. (2015)
*Meloidogyne hapla and Rotylenchulus reniformis*	Secret the CEP-like peptide effector mimics and mediate the nitrogen-demand signaling, nodulation, and lateral root development	Tabata and Sawa (2014), Eves-Van Den Akker et al. (2016), Taleski et al. (2018), and Ota et al. (2020)
*Pseudomonas syringae pv. tomato DC3000*	Regulate ARF7 mediated auxin signaling to induce later root formation	Kong et al. (2020)
*Rhodococcus fascians*	Altered leaf morphology is related with KNOX gene expression and Induce neoplastic, shooty outgrowths *via* regulating mitotic cell division	Manes et al. (2004) and Depuydt et al. (2008, 2009)
*Phytoplasmas effectors reprogram host developmental siganling*		
*Aster Yellows* phytoplasma	Secret effector SAP11, interact with TCP transcription factors; and suppressing NbOMT1 to alter stem and leaf proliferation; alter volatile emissions	Martín-Trillo and Cubas (2010) and Sugio et al. (2011a)
*Aster Yellows* phytoplasma	Secret effector SAP54; Degradation of floral homeotic proteins and MTFs; promotes insect colonization with the RAD23 to alter flower development; cause phyllody	MacLean et al. (2011), Aurin et al. (2020), and Jagdale et al. (2021)
*Aster Yellows* phytoplasma	Secret effector TENGU and down-regulate the auxin-responsive genes to alter plants development	Nagpal et al. (2005), Hoshi et al. (2009), and Minato et al. (2014)
*Wheat blue dwarf* phytoplasma	Secret effector protein SWP11 and target plant proliferation	Wang et al. (2018)
Peanut witches’-broom phytoplasma	Secret effector PHYL1, and interact with MADS domain proteins SEPALLATA3, APETALA1, and CAULIFLOWER to induce flowers morphological changes; prolong vegetative growth	Maejima et al. (2014)
*Candidatus Phytoplasma mali*	Secret effector protein ATP_00189 and target the TCP transcription factors MdTCP24 and MdTCP25 to alter flower development	Janik et al. (2017)
*Jujube* witches’ broom phytoplasma	Secret effector protein SJP1 and SJP2 and target ZjBRC1 that binds to ZjPIN1c/3 to promote the accumulation of indole-3-acetic acid to induce witches’ broom and simulate the lateral bud outgrowth	Zhou et al. (2021)
*Candidatus* phytoplasma	Secret effector protein SAP05 and interact with AtRPN10, and GATA and SPL TFs to prolong vegetative growth disrupte reproductive growth	Furch et al. (2021) and Huang et al. (2021)
*Reprogramming development as an active defense mechanism of host*		
*Pseudomonas syringae*	Arabidopsis shed cauline leaves; HAESA/HAESA-like 2, INFLORESCENCE DEFICIENT IN ABSCISSION, and NEVERSHED were required for the leaf abscission.	Lim and Kunkel (2005), Kissoudis et al. (2016), and Patharkar et al. (2017)
*Peronospora parasitica Pseudomonas syringae Xanthomonas campestris*	Susceptible Arabidopsis plants reduce flowering time and alter aerial branches number on the primary inflorescence	Korves and Bergelson (2003)
*Pseudomonas aeruginosa*	In sensing L-2-amino-4-methoxy-trans-3-butenoic acid (AMB), Arabidopsis blocks seed germination *via* a DELLA-dependent but GA-independent mechanism;	Chahtane et al. (2018)
*Pseudomonas syringae*	Arabidopsis reduces stomatal density to decrease bacteria entry	Dutton et al. (2019)

## 2. Altering host growth and development as a virulence strategy

### 2.1. Pathogen modulates host development *via* manipulating hormone signaling

Phytohormones are multifaceted regulators of plant development and defense. Pathogens can regulate plants’ growth and development as a virulence strategy by mimicking phytohormones or altering plants hormone signaling. Plant-parasitic nematodes colonize and reprogram root cells (Siddique et al., 2015). Cyst and root-knot nematodes penetrate plant roots and migrate to a competent cell in the vascular cylinder to induce a feeding cell complex. Cyst nematodes induce the syncytium by dissolving cell-wall of neighboring cells and fusing their protoplasts (Zhang et al., 2017). Root-knot nematodes recruit the progenitor cell, induce cell enlarge and repeated mitosis without cytokinesis, and eventually form a giant cell (Jagdale et al., 2021). Thus, reprogramming host cell differentiation is an essential virulence strategy for nematode survival. Auxin has been detected in secretions from root-knot nematode, *Meloidogyne incognita* and cyst nematode*, Heterodera schachtii* (Oosterbeek et al., 2021). It is suggested that nematode-secreted auxin at the feeding sites trigger local auxin accumulation and induce cell fate reprogramming (Niebel et al., 1994; Siddique et al., 2015). In addition, nematodes use effectors to manipulate auxin signaling. An effector protein 19C07 secreted by *H. schachtii* can target the Arabidopsis LAX3 auxin import protein to increase the auxin influx onto feeding sites (Lee et al., 2011); LAX3 can trigger the cell wall hydrolysis to stimulate syncytium development and allows the lateral root to emerge (Swarup et al., 2008; Lee et al., 2011). Another effector 10A07 interacts with a plant kinase [interacting plant kinase: (IPK) and the auxin regulator protein INDOLEACETIC ACID-INDUCED16 (IAA16)] in Arabidopsis, preventing it from repressing auxin response genes. Transgenic plant expressed 10A07 displayed stunted shoot sand roots phenotype and more susceptible to nematode infection (Hewezi et al., 2015).

Nematodes also secrete plant peptide hormone (PPH) mimics to shape plant development and facilitate pathogen parasitism (Chen et al., 2015; Ronald and Joe, 2018). Multiple classes of PPH effector mimics have been documented from nematodes including clavata3/embryo surrounding region (CLE)-like, C-terminally encoded peptide (CEP)-like, and inflorescence deficient in abscission (IDA)-like peptides (Ronald and Joe, 2018). CLE-like peptide mimics share sequence homology with plants CLEs, which regulate shoot, root, and vascular meristem maintenance (Lu et al., 2009; Yamaguchi et al., 2016; Ronald and Joe, 2018). Plant A-type CLEs can bind to CLAVATA1 (CLV1)/CLV2 heterodimer and suppress the apical meristem activity and promote cell differentiation (Ito et al., 2006), while B-type plant CLEs can bind to a tracheary element differentiation inhibitory factor (TDIF)-receptor (TDR) and suppress tracheary elements differentiation and promote cell division (Whitford et al., 2008). Nematodes encode both A-type and B-type CLE-like peptide mimics (Replogle et al., 2011; Guo et al., 2017). These CLE-like peptide effectors are secreted and packed into secretory granules and then delivered to the cytoplasm of host root cells through their stylet (Katsir et al., 2011; Mitchum et al., 2012). The peptide mimics then undergoes post-translational modification and interacts with plant CLEs receptor complex, including CLV1, CLV2, and BARELY ANY MERISTEMs and TDR to induce massive cell proliferation and feeding cells formation (Chen et al., 2015; Yamaguchi et al., 2016). CEP-like peptide effector mimics that identified from *Meloidogyne* and CEPs mediate the nitrogen-demand signaling, nodulation, and lateral root development in plants (Taleski et al., 2018). Plant CEPs peptides can move from xylem vessels to the shoots and bind to the leucine-rich repeat receptors, CEPR1 and CEPR2 (Tabata and Sawa, 2014), which induces the CEP downstream 1/2 (CEPD1/2) to upregulate the nitrogen transporter NRT2.1 (Ota et al., 2020). Over-expression of CEP displays phenotype with reduced root cell proliferation and primary root elongation, and increased lateral root development (Ohkubo et al., 2017). Nematode CEPs were also found to upregulate NRT2.1 and reduce primary root length. Eves-Van Den Akker and colleagues suggested that nematodes CEPs limit the expansion of feeding sites to prevent draining excessive nutrient from plants and kill host plants (Eves-Van Den Akker et al., 2016).

Bacterial pathogens also manipulate auxin signaling to alter root development (Kong et al., 2020). Wound caused by emerging lateral roots can be an entry point of bacterial pathogens including *Pseudomonas syringae pv. tomato* strain DC3000 (*Pto* DC3000; Kong et al., 2020). Interestingly, *Pto* DC3000 infection strongly triggered lateral root formation. Auxin response factor 7 (ARF7) and ARF19 are required for the *Pto* DC3000-induced lateral root formation. SA, a key phytohormone against biotrophic pathogens, can suppress lateral root formation, presumably blocking bacteria entrance. Arabidopsis SA deficit mutants show enhanced DC3000-induced lateral root development. ARF7, a well-known regulator of lateral root development, antagonizes the expression of SA marker genes and promotes lateral root development (Kong et al., 2020). These observations indicate an antagonistic interaction between ARF7-regulated auxin signaling and SA signaling in governing lateral root formation, a potential entrance of pathogens into Arabidopsis (Kong et al., 2020). It is speculated that *Pto* DC3000 can manipulate auxin signaling to promote entrance during infection. The virulence factor triggers this developmental change is still unclear (Kong et al., 2020).

Arabidopsis infected by *R. fascians,* a gram-positive phytopathogenic bacterium, displays a spectrum of developmental phenotypes including a narrow lamina, serrated leaf margin, and an uneven leaf surface (Manes et al., 2004; Depuydt et al., 2009). One cellular change associated with these developmental phenotypes is the neoplastic outgrowth in the infected tissue caused by excessive mitotic cell division (Depuydt et al., 2009). *R. fascians* employs multiple strategies to keep infected leaves at an undifferentiated stage. For example, Class-I KNOX genes (KNOTTED-like homeobox, KNAT), required for maintaining the undifferentiated status of cells in the shoot apical meristem, are induced in symptomatic leaves (Depuydt et al., 2008). Constitutional expression of KNOX in plants led to the reduction of gibberellic acids (GA) activity and induction of cytokinin (CK) levels. *R. fascians* infection modulates the plant CK metabolism and activates the CK biosynthesis *via* Arabidopsis response regulators 5/cytokinin 5 (ARR5/CK5) signaling, resulting in small and narrow leaf blades and serrated leaf margins (Depuydt et al., 2008). In addition, *R. fascians* can recruit the CYCLIN D3/RETINOBLASTOMA RELATED (CYCD3/RBR) pathway to stimulate G1-to-S transition and promote proliferation over differentiation (Depuydt et al., 2008, 2009). It is suggested that *R. fascians* infection stimulated the two major cell cycle checkpoints transition, which plays a critical role in symptom development. Consequently, with the cell cycle checkpoints manipulation and hormonal signaling regulation, the infected leaves will reach a state of eternal youth. Although it is unclear how *R. fascians* benefits from undifferentiated cells, studies on age-related disease resistance suggest that pre-mature tissues tend to be more susceptible to pathogen infection (Ficke et al., 2002; Calonnec et al., 2018; Mansfeld et al., 2020). Premature cucumber and strawberries are more susceptible to phytophthora and fungal pathogens (Asalf et al., 2014; Mansfeld et al., 2020). It is reasonable to speculate that *R. fascians* may use multiple strategies to suppress the host ontogenetic resistance by keeping infected leaves at a young stage.

### 2.2. Phytoplasma effectors reprogram host development by targeting conserved transcription factors

Phytoplasmas are a group of obligate phloem bacterial pathogens that can be transmitted among its host plants by insect vectors (Sugio et al., 2011b). Phytoplasmas infected plants display phloem necrosis, witches’ broom, phyllody, and dwarfism (Hogenhout et al., 2008, 2009; Rashid et al., 2018; Omenge et al., 2021). Recent studies revealed that such developmental changes are induced by phytoplasmas effectors (Hogenhout et al., 2008; Hogenhout and Loria, 2008; Sugio et al., 2011a,b; Bertaccini et al., 2019; Furch et al., 2021; Omenge et al., 2021).

Witches’ broom and dwarfism associated with phytoplasma infection can be induced by single effectors. Transient expression of phytoplasma virulence effector, tengu-su inducer (TENGU), secreted by Onion Yellows phytoplasma strain Mild (OY-M), in *Nicotiana benthamiana* phenocopied witches’ broom and dwarfism symptoms (Hoshi et al., 2009). TENGU localizes in parenchyma, meristem tissues, and the apical buds. Transgenic TENGU plants showed downregulated auxin-responsive genes in microarray analysis, including *Auxin/Indole-3-Acetic Acid (AUX/IAA)* family genes *IAA29*, and *IAA7*/*AUX2*, small auxin-induced RNA (SAUR) family genes *SAUR_AC1*, and *Gretchen Hagen 3 (GH3)* family genes *GH3.5*/*WES1*, which suggested that TENGU can inhibit auxin-related signaling and alter plants development (Hoshi et al., 2009). In addition, TENGU can interfere with the jasmonic acid biosynthesis to cause plant sterility without floral malformations (Minato et al., 2014). Auxin response factors, ARF6 and ARF8, promote floral maturation by activating Jasmonic Acid (JA) synthesis or by decreasing JA degradation (Nagpal et al., 2005). The expression of *ARF6* and *ARF8* were significantly decreased in both transgenic TENGU-plants and phytoplasma-infected plants. Consequently, JA level was decreased in TENGU-transgenic buds (Minato et al., 2014). Thus, TENGU hijack multiple nodes of auxin signaling to manipulate flower development.

Phytoplasma effectors also contribute to the phyllody phenotype (Bertaccini, 2007). The molecular mechanism of flower abnormalities induced by phytoplasma infection was first documented in tomatoes (Pracros et al., 2006; Sugio et al., 2011a,b). *Stolbur* phytoplasma infected tomato showed virescence, phyllody, sepal hypertrophy, and aborted reproductive organs (Pracros et al., 2006). Expressing SAP54, an effector of Aster Yellows phytoplasma strain Witches’ Broom (AY-WB), was sufficient to induce phyllody in Arabidopsis (Aurin et al., 2020). Further studies showed that SAP54 alters the host plant reproductive and floral development by degrading a group of type II MADS-domain transcription factors (MTFs) regulating the floral transition and floral organ development (Jagdale et al., 2021). *Arabidopsis* radiation-sensitive-23 (RAD23) family proteins, RAD23C, and RAD23D, physically interact with SAP54 and are required for the degradation of host MTFs and phytoplasma-induced phyllody (MacLean et al., 2014). Aster leafhopper *Macrosteles quadrilineatus* has oviposition preference for plants with green leaf-like flowers induced by SAP54 (MacLean et al., 2011; Aurin et al., 2020). It is proposed that SAP54-induced phyllody facilitates the transmission of AY-WB.

Another phytoplasma AY-WB secreted effector protein 11 (SAP11) alters shoot proliferation and leaf shape changes (Pecher et al., 2019). Arabidopsis transgenic plant expressing SAP11 displays large curly leaves and overproduces axillary stems (Martín-Trillo and Cubas, 2010; Sugio et al., 2011a,b). SAP11 interacts with and destabilizes Arabidopsis TCP [TEOSINTE BRANCHED, CYCLOIDEA, PROLIFERATING CELL NUCLEAR ANTIGEN FACTOR1 (PCF1), and PCF2] transcription factors including TEOSINTE BRANCHED1, PROLIFERATING CELL FACTORS 1 and 2, TCP2, TCP7, TCP13, and CYCLOIDEA transcription factors (Martín-Trillo and Cubas, 2010; Sugio et al., 2011a,b). TCP transcription factors function to suppress excessive growth of leaf and shoot branching during normal growth (Dhaka et al., 2017). Although it is unclear how the altered leaf morphology and branching number benefit phytoplasma, SAP11 can also suppress JA biosynthesis to enhance the survival and reproduction of the insect vectors (Sugio et al., 2011a). JA is a major phytohormone that is involved in the defense against the AY-WB leafhopper vector *M. quadrilineatus* and *M. quadrilineatus* can produce about 60% more progeny on AY-WB-infected plants with the decreased JA expression (Sugio et al., 2011a). The transgenic Arabidopsis plants expressing SAP11 accumulate less JA. The destabilized Arabidopsis TCPs by SAP11 can reduce the lipoxygenase (LOX) genes expression, which leads to reduced JA synthesis (Sugio et al., 2011a). SAP11 homologs are reported to alter the development as well in other plant species including maize, wheat, and coconut (Pecher et al., 2019). Maize Bushy Stunt Phytoplasma (MBSP), a SAP11 homolog that mainly infects maize, can also bind the helix–loop–helix region of the TCP domain, and destabilize the TEOSINTE BRANCHED 1/CYCLOIDEA (TB1/CYC) TCPs. SAP11 homolog of MBSP can induce axillary branching like the AY-WB SAP11, while preventing the female inflorescence development and inducing the tassels feminization (Pecher et al., 2019). Since all the disease symptoms induced by the phytoplasmas generate more young and green tissues, phytoplasma insect vectors have preference on the young and green tissues for feeding and laying eggs (Hogenhout et al., 2008; Hogenhout and Loria, 2008). It is speculative that phytoplasma induced developmental changes are part of the pathogen’s virulence strategy to enhance their fitness. Phytoplasmas generates effectors that target plant development processes resulting in generating more young vegetative tissue (witches’ broom and phyllody, etc.) or prolonging the plant host lifespan. The young vegetative tissue attracts more insect sectors, which helps the transmission of phytoplasmas. Phytoplasmas, as a biotrophic pathogen, benefits from the prolonged lifespan of its plant host, which increases the phytoplasmas fitness. Another speculation is that phytoplasmas modulate the plants’ development to regulate the hormone production or alter the plants defense hormone signaling like JA, thus increasing the susceptibility of the host, which improves fitness (Sugio et al., 2011b).

## 3. Reprogramming development as a host defense mechanism

During host-pathogen interaction, plants may actively reprogram their growth and development as a defense strategy. Cauline leaves of Arabidopsis were shed after infected by *Pto* DC3000 with a functional type III secretion, which is proposed as a defense mechanism to limit pathogen invasion (Patharkar et al., 2017). Leaf abscission as an active defense only occurs when bacteria physically contact the cauline leaf abscission zone. Regulators of abscission in normal development such as *HAESA*/*HAESA-like 2*, *INFLORESCENCE DEFICIENT IN ABSCISSION*, and *NEVERSHED* were required for the leaf abscission under bacterial infection, indicating that a normal developmental machinery was reprogrammed during plant-microbe interaction. SA may serve as a link between bacterial sensing and leaf shedding since SA-deficient mutants fail to shed cauline leaves upon infection (Patharkar et al., 2017). Several other plant species show abscission of infected organs in response to pathogens as well. For example, tomatoes shed their leaves in response to *P. syringae* and *powdery mildew* infection (Lim and Kunkel, 2005; Kissoudis et al., 2016), suggesting that shedding infected organs might be a common defense mechanism *via* reprogramming a developmental process.

Plants can also change the timing of their developmental progression as a defense strategy. Arabidopsis seed germination was arrested in the presence of *Pseudomonas aeruginosa* (Chahtane et al., 2018). l-2-Amino-4-methoxy-trans-3-butenoic acid (AMB) is a non-proteinogenic amino acid toxin for prokaryotes and eukaryotes produced by *P. aeruginosa*. Interestingly, upon the detection of AMB, plants induced DELLA proteins in seeds, leading to a DELLA-dependent but GA-independent arrest of seed germination. In this process, germination repressor ABI5 was also over-accumulated (Chahtane et al., 2018). Since arrested seed germination has no clear benefit to the bacterial pathogen, it is speculated that the delay of germination in the presence of *P. aeruginosa* is to avoid deleterious seedling infection.

In another case, accelerated flowering together with increase branches were observed in Arabidopsis challenged with three different pathogen species including two bacterial pathogens, *P. syringae* and *Xanthomonas campestris*, and an oomycete, *Peronospora parasitica* (Korves and Bergelson, 2003). Korves suggested that these similar developmental changes in susceptible Arabidopsis were the general developmental responses to pathogen infection that may affect tolerance of and/or resistance to disease (Korves and Bergelson, 2003). It is noteworthy that plants gain age-related resistance (ARR) during shoot maturation including flowering (Wilson et al., 2013; Lyons et al., 2015; Hu and Yang, 2019). For example, *Nicotiana tabacum* was found to gain resistance to pathogen *P. parasitica* upon flowering (Wyatt and Kuc, 1992). Thus, pathogen-induced flowering could be a defense mechanism to activate ARR.

Stomata is one of the natural openings for many pathogens to enter plant hosts (Melotto et al., 2006, 2008). The regulation of stomata opening is a battlefield of host and pathogens. On one side, plants motivate immune responses to close stomata upon pathogen detection, while pathogens use hormone mimics or effector proteins to open stomata (Gimenez-Ibanez et al., 2014). Interestingly, the battle is not only limited to mature stomata but extends to the early stage of stomata differentiation. Upon *P. syringae pv. tomato* (*Pto* DC3000) infection, Arabidopsis reduces stomatal density by 20% in the subsequently developed new leaves with many epidermal pavement cells (Dutton et al., 2019). It is speculated that such a reduction serves as a defense mechanism to decrease bacteria entry. The mechanism of pathogen-induced reduction of stomatal density remains unknown. Dutton suggested that the stomatal reduction is possibly mediated by components of plant immune system such as the AZI protein, a potential systemic signal, flagellin receptor, and SA biosynthesis (Dutton et al., 2019). Given that *Pto* DC3000 infection could trigger lateral root emergence (Kong et al., 2020), plant host and pathogens are in an arms race to create or limit entrance.

## 4. Summary marks and future direction

**Figure 1:**
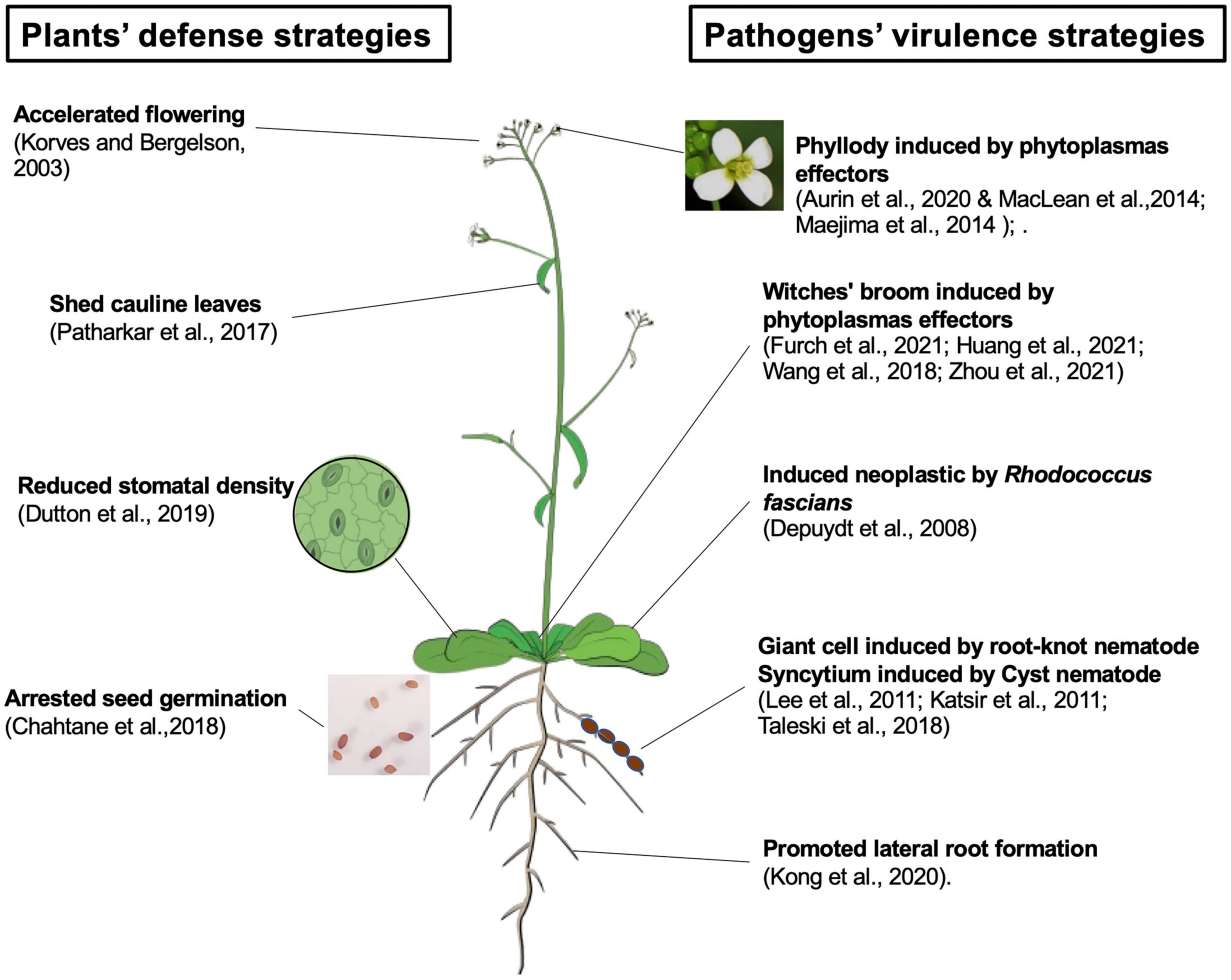
Pathogens triggered changes in plants development. Pathogen-triggered development changes in plants can be results of active defense strategies, including accelerating flowering time, shed its cauline leaves, reduced the stomatal density, and arrest seed germination. Pathogens’ virulence strategies also target host development. Such changes can be achieved by effectors, such as phytoplasma effector induced phyllody and witches’ broom, or hormone mimics including neoplastic growth induced by *Rhodococcus fascians* and giant cell/syncytium formed during nematode infection. The Arabidopsis model was cited from Illustrations, Plant (2017): Shoot illustrations. Figshare. Collection. https://doi.org/10.6084/m9.figshare.c.3701035.v13.

Here, we summarize the current understanding of pathogen-triggered changes in plant development (Figure 1). Recent progress in studying pathogens’ virulence strategies, such as function of effectors, have helped to distinguish whether altered host development is a consequence of pathogen virulence or active host defense. However, it is still challenging to elucidate how a pathogen-induced developmental change benefits the causal agent. A deeper investigation of pathogen physiology and life cycle is valuable to fill the gap. Although our understanding of the plant development-defense tradeoff is accumulating (Huot et al., 2014; He et al., 2022), further dissecting the molecular details of host development-defense crosstalk will contribute to understanding how developmental changes can be used as a defense tool.

## Author contributions

FK and LY conceptualized and wrote the manuscript. All authors contributed to the article and approved the submitted version.

## Funding

This project is supported by NIH R35GM143067 to LY.

## Conflict of interest

The authors declare that the research was conducted in the absence of any commercial or financial relationships that could be construed as a potential conflict of interest.

## Publisher’s note

All claims expressed in this article are solely those of the authors and do not necessarily represent those of their affiliated organizations, or those of the publisher, the editors and the reviewers. Any product that may be evaluated in this article, or claim that may be made by its manufacturer, is not guaranteed or endorsed by the publisher.
